# Changing housework, changing health? A longitudinal analysis of how changes in housework are associated with functional somatic symptoms

**DOI:** 10.3402/ijch.v75.31781

**Published:** 2016-06-30

**Authors:** Evelina Landstedt, Lisa Harryson, Anne Hammarström

**Affiliations:** 1 Epidemiology and Global Health, Department of Public Health and Clinical Medicine, Umeå University, Umeå, Sweden; 2 Department of Sociology, Umeå University, Umeå, Sweden

**Keywords:** domestic work, functional somatic symptoms, embodiment, gender theory, longitudinal analysis

## Abstract

**Aim:**

The aim of this study was to analyse how changes in housework over the course of adulthood are related to somatic health in Swedish men and women.

**Methods:**

Data were drawn from 2 waves of the Northern Swedish Cohort Study, response rate 94.3%, N=1,001. A subsample of cohabiting individuals was selected (n=328 women, 300 men). Outcome variable was functional somatic symptoms (FSS) at age 42. Associations were assessed in multivariate general linear models with adjustment for confounders and somatic health at age 30.

**Results:**

Housework is primarily performed by women, and women's responsibility for and performance of housework increased from ages 30 to 42. These changes were associated with elevated levels of FSS at age 42 in women. Men reported considerably lower responsibility for and performed less housework compared with women, the load of housework for men does not change substantially from ages 30 to 42 and no associations with FSS were identified.

**Conclusions:**

The gendered division of housework means that women are particularly exposed to a heavy workload. Women's responsibility for and performance of housework increase between ages 30 and 42 and this threatens to be embodied in the form FSS. We conclude that housework should be considered an important source of stress in addition to that from waged work and that a deeper understanding of the links between housework and health requires a gender theoretical analysis.

Existing evidence shows links between housework and health among women and men (1–3). Generally, a higher load of housework are associated with lower self-rated health among women (4), whereas satisfaction with the division of housework is associated with reduced risks for sickness absence among men (2). Cohabiting women and men with unequal responsibility for the housework have higher risks for psychological distress (1). Overall, previous research on the health consequences of housework has primarily focused on the psychosocial aspects, with a lack of studies including both women and men that explore somatic health status (5,6). There is also a knowledge gap regarding how changes in housework over time are related to somatic health status. One of the limitations in previous studies adopting a life course perspective on housework is that they generally focus on specific events such as marriage and childbirth, rather than investigating how change in housework across time is related to health status (7). For instance, the birth of a child often leads to increased housework, and dominant norms upholding the unequal division of labour, whereby women take up the greater burden than men, become more common (8,9). One of the few longitudinal studies within the field suggests that more the responsibility for housework and childcare, less the feeling of fairness and satisfaction among women (10). Explorations of possible health consequences from change in housework across time are missing.

Together with the other Nordic countries, Sweden represents a dual-earner welfare model with a strong political support for gender equality in family and working life (11). This model has been found to promote better health than less egalitarian models (1,11,12). However, the normative and cultural expectations of gender practices imply symbolic constructions of childcare and housework as women's work and a way for women to show love for their family (13). For example, although the amount and type of both paid and unpaid work changes across life depending on, for example, cohabitation, marriage, childbirth and separation (9), heterosexual cohabiting Swedish women still have the main responsibility for the unpaid housework (14). The trend towards a more gender equal division of unpaid work at home in industrial countries is primarily due to a reduction of the hours women spend doing this work (9,14). These gendered patterns are often referred to as the gender division of labour, which means that women and men are exposed to partially different environments and responsibilities, which in turn can be associated with health either negatively or positively (15,16). The workload from combined waged and housework seems to lead to an increased risk of health problems (17), and the double burden of paid and unpaid work becomes a health risk (18,19).

To understand the complex systems of how the social process of housework interact with bodily expressions of health, we use an epidemiological framework of embodiment that emphasizes the integration of soma, psyche and society (20,21). According to Krieger, embodiment represents a biological incorporation of the material and social world, and how bodies change with environmental and behavioural factors, such as social gender relations and gendered practices of housework (20,21). In this study, functional somatic symptoms (FSS) represent the possible bodily response to exposure to housework. We use FSS to signify a spectrum of self-perceived bodily complaints that are experienced as a transformation from normal health status to often-unexplained somatic symptoms (22).

The aim of this study was to analyse how changes in housework over the course of adulthood were related to FSS among women and men.

## Methods

### Sample and data collection

Data were drawn from the Northern Swedish Cohort, which consists of all pupils (n=1,083; 506 girls and 577 boys) who studied in their last year of compulsory school in a medium-sized Swedish industrial town in 1981. The questionnaire included questions concerning school, employment, socio-economic conditions and health. Participants have subsequently filled in a similar questionnaire in 1983, 1986, 1995 and 2007. The response rate (in relation to those still alive in the original cohort) was 94% in 2007 (23). This study is based on data from ages 30 years (1995) to 42 years (2007) and includes only those who, at both waves, lived with a partner and/or was married (n=628, 52.2% women).

### Measures

#### Outcome

*FSS* (for ages 30 and 42) were measured through 10 self-reported somatic symptoms: headache or migraine; other stomach ache (than heartburn, gastritis or gastric ulcer); nausea; backache, hip pain or sciatica; general tiredness; breathlessness; dizziness; overstrain; sleeping problems; and palpitations. Items were coded “No, never” (0); “On and off” (1); and “Often/all the time” (2). The scale was computed as the mean of the 10 item values (range 0–2) and shows acceptable psychometric properties, including, for example, factor structure, internal consistency and invariance in factor structure over time (24).

#### Independent variables – main exposure

*Responsibility for housework* (for ages 30 and 42) was measured with the question, “How much of the responsibility for the housework do you take?” The answer alternatives were “none,” “less than half,” “more than half” and “all.”

*Change in responsibility for housework* was created by the variables of responsibility for housework at ages 30 and 42**. To reduce the number of categories and to increase the statistical power in the analysis, responsibility for housework was dichotomized into 2 groups at ages 30 and 42: low responsibility (none, less than half or half) and high responsibility (more than half or all). The combined variable, “change in responsibility for housework,” was categorized into 4 groups:

- Low responsibility at ages 30 and 42
- High responsibility at age 30 and low responsibility at age 42
- Low responsibility at age 30 and high responsibility at age 42
- High responsibility at ages 30 and 42

*Household work time* (ages 30 and 42) was measured as the number of hours per week spent on housework duties such as cooking, washing and cleaning. The answer alternatives were “no time,” “<1 hour,” “1–3 hours,” “4–7 hours,” “8–14 hours,” “15–21 hours,” “22–35 hours” and “more than 35 hours.” These items were used to construct 2 variables reflecting somewhat different aspects of change in the amount of time spent on housework: “change in amount of housework,” which indicates stability or change in categories of low/medium amount or high amount, and “change in time spent on housework,” which reflects change or not regardless of initial number of hours per week spent on housework.

*Change in amount of housework* was created by the variables of time in housework at ages 30 and 42**. In order to reduce the number of categories and to increase the statistical power in the analysis, time in housework was dichotomized into 2 groups at ages 30 and 42: low amount (0–14 hours/week) and high amount (>14 hours/week). The combined variable “change in housework time” was categorized into 4 groups:

- Low amount at ages 30 and 42
- High amount at age 30 and low amount at age 42
- Low amount at age 30 and high amount at age 42
- High amount at ages 30 and 42

*Change in time spent on housework* is the variable that was computed by calculating the difference in hours of housework per week (see description above) between ages 30 and 42 and then recoding the numeric variable into “no change,” “decrease” and “increase.”

Given the different approach in how these 2 time-related measures account for time spent on housework at age 30, the variables complement each other.

### Covariates

*Living with children at age 30* was measured as if the participants were living with children all or some of the time (0) or were not living with children (1). Living with children was assumed to be linked to elevated levels of housework and, therefore, included as a possible confounder in the analyses.

*Time in paid work at age 30* was measured as the number of hours in paid work (0–82 hours) per week. Logically, the more hours spent on paid work, the less time for housework. However, as shown in the introduction this relationship is complex and highly gendered (1).

*Occupational status* (age 30) was measured with occupation level on the basis of the Swedish SEI classification (25): upper white-collar workers including self-employed (0), lower white-collar (1) and blue-collar workers (2).

### Ethics statement

The Regional Ethical Review Board in Umeå, Sweden, has approved this study.

### Statistical analysis

Between-group analyses were performed using independent sample t-tests and ANOVAs. Crude and multivariate general linear models (GLMs) were performed for each housework exposure in relation to FSS. Given the distinct gender pattern in both the outcome and the main exposures, the regression analyses were conducted separately for women and men. Adjustments were made for the following variables at age 30: FSS, living with children, average number of hours spent on paid work per week and occupational status. All statistical analyses were performed using PASW Statistics 22 with a significance level at 0.05.

## Results

Table I displays the distribution of variables included in the study. The distribution of the housework variables was highly gendered with women reporting more responsibility for and spending more time doing housework than men. For men, the pattern of change in amount of housework is very similar to that of change in responsibility: nearly 9 out of 10 men remained in the stable low category. Every second woman remained in a stable high responsibility category. They were also more likely than men to have shifted from high to low responsibility and vice versa. With regard to change in amount of housework, both the shifts (from high to low amount and vice versa) and the stable high scenario were more common in women than men. When only looking at change in the number of hours spent on housework, one-quarter of all participants, regardless of gender, reported no change, whereas between 30 and 44% reported decrease or increase.

**Table I:** Description of main exposure and health outcomes and difference between women and men

	Total	Women	Men	
		
	N (%)	N (%)	N (%)	p^a^
Categorical variables				
Housework responsibility at age 42				<0.001
No	2 (0.3)	0 (0)	2 (0.7)	
Less than half	131 (20.9)	11 (3.4)	120 (40.0)	
Half	249 (39.6)	102 (31.1)	147 (49.0)	
More than half	219 (34.9)	191 (58.2)	28 (9.3)	
All	27 (4.3)	24 (7.3)	3 (1.0)	
Total	628	328	300	
Change in housework responsibility between ages 30 and 42				<0.001
Low–low	307 (50.1)	59 (18.1)	248 (86.4)	
High–low	81 (13.2)	56 (17.2)	25 (8.7)	
Low–high	62 (10.1)	53 (16.3)	9 (3.1)	
High–high	163 (26.6)	158 (48.5)	5 (1.7)	
Total	613	326	287	
Housework time at age 42				<0.001
< 1 hour	23 (3.6)	5 (1.5)	18 (6.0)	
1–3	95 (15.2)	18 (5.5)	77 (25.9)	
4–7	167 (26.8)	81 (24.8)	86 (29.0)	
8–14	213 (34.1)	129 (39.4)	84 (28.3)	
15–21	94 (15.1)	66 (20.2)	28 (9.4)	
>22–35	32 (5.1)	28 (8.6)	4 (1.3)	
Total	624	327	297	
Change in amount of housework between ages 30 and 42				<0.001
Low–low	421 (68.6)	173 (53.9)	248 (84.6)	
High–low	69 (11.2)	56 (17.4)	13 (4.4)	
Low–high	88 (14.3)	59 (18.4)	29 (9.9)	
High–high	36 (5.9)	33 (10.3)	3 (1.0)	
Total	614	321	293	
Change in time spent on housework				0.271
No change	162 (26.4)	85 (26.5)	77 (26.3)	
Decrease	202 (32.9)	114 (35.5)	88 (30.0)	
Increase	250 (40.7)	122 (38.0)	128 (43.7)	
Total	614	321	293	
Living with children at age 30				<0.001
Yes	442 (70.5)	257 (78.4)	185 (61.9)	
Total	627	328	299	
Occupational status at age 30				ns
1	345 (55.4)	180 (55.0)	165 (55.7)	
2	38 (6.1)	26 (8.0)	12 (4.1)	
3	240 (38.5)	121 (37.0)	119 (40.2)	
Total	623	327	296	
Continuous variables	M (SD)	M (SD)	M (SD)	
Paid work hour/week at age 30	36.21 (14.1)	32.36 (13.6)	40.10 (13.47)	<0.001
Functional somatic symptoms at age 42	0.39 (0.30)	0.43 (0.31)	0.34 (0.28)	<0.001
Functional somatic symptoms at age 30	0.36 (0.29)	0.40 (0.29)	0.31 (0.27)	<0.001

a Between-group differences calculated with independent sample t-test for continuous variables and χ^2^-test for categorical variables.

Seventy percent of the sample lived with children at age 30, and men spent more time in paid work than did women. More than half of the sample were white-collar workers including self-employed, 6% lower white-collar workers and 4 out of 10 as blue-collar workers. As shown in Table I, women reported higher levels of FSS than men at both ages 30 and 42.

Table II presents how levels of FSS were distributed between the categories of change in housework. With regard to change in responsibility for housework, the analysis of the entire sample shows a difference in which FSS was least common in the low stable group and most common in the group of decreased responsibility from high to low. However, gender-separate analyses showed no difference in FSS depending on whether the participants remained in the altered or stable groups regarding responsibility. This variable was therefore not included in the regression analyses. Table II further shows that changes from less to more hours in housework were associated with higher levels of FSS at age 42, compared to all other categories including the high stable group. This finding was, however, only valid for women.

**Table II:** Change in housework responsibility and amount of housework in relation to functional somatic symptoms

	Functional somatic symptoms, age 42		
	
	Total	Women	Men
	
	M (SD)	M (SD)	M (SD)
Change in housework responsibility			
Low–low	0.34 (0.28)	0.36 (0.28)	0.33 (0.28)
High–low	0.45 (0.32)	0.48 (0.33)	0.38 (0.29)
Low–high	0.41 (0.32)	0.44 (0.32)	0.28 (0.24)
High–high	0.43 (0.31)	0.44 (0.31)	0.26 (0.18)
p	0.001; F=5.22	0.22; F=1.48	0.74; F=0.42
Change in amount of housework			
Low–low	0.37 (0.29)	0.42 (0.30)	0.34 (0.28)
High–low	0.37 (0.27)	0.38 (0.26)	0.31 (0.33)
Low–high	0.48 (0.36)	0.54 (0.38)	0.36 (0.27)
High–high	0.41 (0.30)	0.41 (0.30)	0.40 (0.35)
p	0.01; F=3.65	0.03; F=3.14	0.92; F=0.17
Change in time spent on housework time			
No change	0.38 (0.30)	0.38 (0.29)	0.37 (0.31)
Decrease	0.38 (0.29)	0.40 (0.27)	0.36 (0.30)
Increase	0.40 (0.32)	0.50 (0.35)	0.30 (0.25)
p	0.70	0.009; F=4.74	0.18

Between values calculated with ANOVA.

Results from the GLMs are found in Table III (women) and include only variables for which there were between-categories differences in FSS. In women, the crude analysis (Model 1) confirmed that, in comparison with the reference category (stable low or no change), the increased amount and time of housework between ages 30 and 42 was associated with elevated levels of FSS. This association remained after adjusting for FSS at age 30 (Model 2) as well as having children in the household, the number of hours spent on paid work and occupational status (Model 3). Detailed results from post hoc tests are available upon request. No statistically significant associations were identified among men (Table IV).

**Table III:** Change in amount of housework and time spent on housework between ages 30 and 42 in relation to functional somatic symptoms among women, age 42 (GLM)

	Women n=321								
	
	Model 1			Model 2			Model 3		
			
	B	SE	p	B	SE	p	B	SE	p
Change in amount of housework									
Low–low	Ref			Ref					
High–low	−0.03	0.05	0.48	−0.03	0.04	0.50	−0.03	0.05	0.45
Low–high	0.13	0.05	0.008	0.11	0.04	0.007	0.12	0.04	0.003
High–high	−0.004	0.06	0.95	−0.02	0.05	0.77	0.01	0.06	0.97
R2	0.03			0.27			0.29		
Change in time spent on housework									
No change	Ref.			Ref.			Ref.		
Decreased	0.02	0.04	0.59	−0.01	0.04	0.82	0.001	0.04	0.98
Increased	0.12	0.04	0.006	0.07	0.04	0.06	0.09	0.04	0.02
R^2^	0.03			0.27			0.28		

GLM, general linear model.

Model 1. Crude model.

Model 2. Adjusted for FSS at age 30.

Model 3. Adjusted for the following variables at age 30: FSS, living with children, hours in paid work per week and occupational status.

**Table IV:** Change in time in housework and functional somatic symptoms among men, age 42 (GLM)

	Men								
	
	Model 1			Model 2			Model 3		
			
	B	SE	p	B	SE	p	B	SE	p
Change in amount of housework									
Low–low	Ref			Ref.			Ref.		
High–low	−0.03	0.08	0.73	−0.03	0.07	0.66	−0.04	0.08	0.63
Low–high	0.03	0.06	0.64	−0.02	0.05	0.76	−0.03	0.05	0.57
High–high	0.06	0.17	0.79	−0.08	0.15	0.61	−0.09	0.15	0.55
R^2^	0.002			0.23			0.24		
Change in time spent on housework									
No change	Ref.			Ref.			Ref.		
Decreased	−0.01	0.04	0.83	−0.001	0.04	0.98	−0.01	0.04	0.90
Increased	0.07	0.04	0.11	−0.03	0.04	0.36	−0.05	0.04	0.25
R^2^	0.01			0.23			0.24		

GLM, general linear model.

Model 1. Crude model.

Model 2. Adjusted for FSS at age 30.

Model 3. Adjusted for the following variables at age 30: FSS, living with children, hours in paid work per week and occupational status.

## Discussion

Although previous research demonstrates associations between level and responsibility for housework and health among women and men (1–3), few studies have explored how longitudinal changes in housework are related to changes in bodily expressions of health in women and men. The main findings of this study show that not only is housework predominately performed by women but also women's responsibility for and performance of housework increases from ages 30 to 42. These changes are associated with elevated levels of FSS regardless of previous FSS, amount of paid work, occupational status and the presence of children in the household. In contrast, men have considerably lower responsibility for and perform less housework compared to women and do not change their load of housework substantially from ages 30 to 42.

Compared to men, women changed their responsibility for and the amount of housework between ages 30 and 42 to a much greater extent. There was no gender pattern in whether the time spent on housework had decreased, increased or remained stable. However, this measure does not account for the level at age 30, that is, no change can mean high load at both ages 30 and 42, or vice versa.

This is a time of life when many people start a family, and research shows that the transition into parenthood tends to increase the amount of housework for women, whereas men's housework is more stable across parenthood (9). The results indicate that the gendered organization of unpaid work at home represents a gender structure upheld by socially constructed positions and norms (13). It is likely that the expectations of women as mainly responsible for the domestic sphere are reflected in the unequal distribution and change of housework (26). Our study suggests that this situation constitutes a risk for women's physical health not only in the present but also over time. Housework, as a part of family relations, is also deeply imbued with embodied interactions and practices. For example, the fact that women more often take care of men rather than the reverse is intrinsically embodied (27). Women's higher risk of FSS may, therefore, be an embodied consequence of the gendered division housework (20).

Our study indicates that men's physical health does not seem to be affected negatively by an unequal division of housework. However, it should be noticed that we do not know whether men's health would be affected in the same way as that of women, in the same situation, because a high workload of hours in housework, as well as changes over time, is much rarer among men than among women. Nevertheless, time spent on housework in relation to psychological distress has previously been investigated within the same population (the Northern Swedish Cohort), without finding any significant associations among either women or men (1). These contradictory results indicate that the burden of performing housework can be difficult to capture through established mental health measures, although it seems to leave its marks in women's bodies (20). From a public health perspective, measuring the time spent on housework is therefore a highly relevant way of capturing bodily health expressions of gender practices in everyday life.

An unexpected result is that decreased responsibility for housework (from higher to lower) did not reduce the level of FSS (Table II). In contrast, those reporting decreased responsibility had higher level of FSS in the total population. Although these results became insignificant in the gender separate analyses, it seems they mainly represent women's situation as women, to a greater extent, have changed their level of housework responsibility. One possible explanation might be that, for women, breaking expected genders norms of housework (in this case, reducing the amount) implies strain on the individual. A similar argument was put forward in a previous Swedish study; being the pioneer of breaking societal gendered norms can be stressful (28). However, even if norm-breaking practices in housework might impact health negatively in the short run, it is well known that gender equality has positive health consequences for both women and men in the long run (12). When new gender relations expand and gain general acceptance, the initial negative health consequences of changes in gender relations will be positive and lead to reduced inequalities in health (29).

### Strengths and limitations

The strengths of the study include, for example, prospective cohort material, low attrition, the sample being representative of the Swedish population (23) and a well-evaluated measure of FSS (24). The findings represent cohabiting individuals rather than couples and the results should be interpreted as patterns at the population level. Also, 7 of 10 already had children at age 30, which indicates that the initial level of housework most likely was fairly high. As the focus in this study was on unpaid work in everyday life, we only included a daily basis for housework and not unpaid work, such as gardening, car care and restoration work. There were problems of statistical power in the analyses of men because of few cases in the high–high categories. Hence, we cannot be sure that the health response of changes in housework in men is dissimilar to that of women. This needs to be scrutinized in future studies.

## Conclusions

The gendered division of housework means that women are particularly exposed to a heavy workload. Women's responsibility for and performance of housework also increase across adulthood, and this situation threatens to be embodied in the form of elevated levels of FSS. In contrast, men have a considerably lower and unchanged load of housework that does not seem to be related to their FSS across time. We conclude that housework should be considered an important source of stress in addition to that from waged work and that a deeper understanding of the links between housework and health requires a gender theoretical analysis. This should be acknowledged in social policy and public health interventions.
